# Glycome dynamics in T and B cell development: basic immunological mechanisms and clinical applications

**DOI:** 10.1016/j.it.2023.06.004

**Published:** 2023-08

**Authors:** Manuel M. Vicente, Eduarda Leite-Gomes, Salomé S. Pinho

**Affiliations:** 1 i3S – Institute for Research and Innovation in Health, University of Porto, 4200-135 Porto, Portugal; 2 Graduate Program in Areas of Applied and Basic Biology (GABBA), School of Medicine and Biomedical Sciences (ICBAS), University of Porto, 4050-313 Porto, Portugal; 3 School of Medicine and Biomedical Sciences (ICBAS), University of Porto, 4050-313 Porto, Portugal; 4 Faculty of Medicine, University of Porto, 4200-319 Porto, Portugal

## Abstract

The glycosylation profile of mammalian mature T and B cells contributes to the regulation of cellular function in immune responses.
The glycome of developing T and B cells shows dynamics across maturing stages.
Interference in glycosylation pathways is known to lead to reprogramming of T and B cell developmental processes, often resulting in mature population defects.
The relationship between glycans in T and B cell development and disease susceptibility is a promising novel research avenue.

## Significance

Glycans are key regulators of lymphocyte biology, highlighted by their prominent role along each T and B cell developmental stage. Further research is needed to clarify the precise contribution of these sugar chains in developmental processes and checkpoints and how this can associate with disease susceptibility for various conditions.

## Teaser

Glycans cover the surfaces of all mammalian cells through a process called glycosylation. Nearly all proteins and receptors that integrate the intricate series of co-stimulatory/inhibitory pathways of the immune system are glycosylated. Growing evidence indicates that the development of the immune system at the origins of T and B cell development is tightly regulated by glycosylation. In this opinion, we hypothesize that the glycome composition of developing T and B cells is developmentally regulated. We discuss how glycans play fundamental roles in lymphocyte development and how glycans early define T and B cell functionality in multiple aspects of adaptive immunity. These advances can provide opportunities for the discovery of novel disease factors and more effective candidate treatments for various conditions.

## Cracking the sugar code of T and B cells

Glycans, or carbohydrates, are present in virtually all cellular surfaces and are important regulators of the mammalian immune system (Box 1) [1]. Not surprisingly, T and B cells contain a dense coat of surface glycans (glycocalyx) that tightly regulate activity and function [2., 3., 4., 5.]. The composition of glycan structures within a certain cell or tissue (its glycome) contains an important amount of biological information. Glycans have been reported to be essential regulators of T and B cells [3,5]. Moreover, the fundamental role of glycans in immunity is vast, spanning from their impact at early stages of T and B cell development, differentiation, and selection, as well as for the B cell production of antibodies and for defining the pro- or anti-inflammatory properties of T cells. In fact, by regulating immune cell-fate decisions, activity, function, as well as early developmental processes in the thymus [2] and bone marrow [6,7], protein glycosylation has been implicated in most cellular processes of humoral and adaptive immunity. Early in the 1970s and 1980s, prosperous times for modern immunology, the peanut agglutinin lectin (PNA), recognizing carbohydrate sequence Gal-β(1-3)-GalNAc, was shown to differentially bind immature and mature thymocytes in mice [8]. Moreover, the binding of PNA to germinal center (GC) B cells became a standard method to identify these cells [9]. These studies represent early highlights of the relevance of glycans in T and B cell development. Recent studies further demonstrated that the glycome composition of human and murine thymocytes is dynamic along different maturation stages, reigniting the hypothesis that this glycosignature is developmentally regulated [10]. We propose that the regulation of glycan profiles is one of the mechanisms enabling normal developmental transitions that, for T and B cells, ensure proficient cellular populations driving adaptive and humoral immunity. In the next sections, we discuss relevant work that supports this view, further examining the consequences of a glycan switch in T and B cell development and function and the implications for disease. We present a brief guided tour through each T and B cell developmental step that focuses on the prominent role of glycans at the origins of humoral and adaptive immunity. The impact of glycosylation upon T and B cell activation is manyfold but this opinion article is focused on the role of glycans as early as in lymphocyte development and function.

Box 1 **Glycosylation pathways**

Glycosylation is a major co- and post-translational modification process responsible for the attachment of glycans to proteins, lipids, or other saccharides. It is mediated by the coordinated action of a collection of different glycosyltransferase and glycosidase enzymes that act in the endoplasmic reticulum (ER) and Golgi apparatus of essentially all cells. This process occurs in a stepwise manner, where most of the steps depend on enzyme activity and location within organelles, amounts of gene transcription, and substrate availability [97]. Glycoconjugates are primarily defined according to the nature of their nonglycosidic part, as well as the linkage between them [98]. In higher vertebrates, within the major glycoconjugates, glycoproteins consist of proteins that carry *N*- and/or *O*-glycosidically linked carbohydrate chains. *N*-glycosylation is initiated by the addition of a sugar to the nitrogen atom of an asparagine residue of a protein in the consensus motif Asn-X-Ser/Thr, where X is not a proline. *O*-glycosylation occurs on serine or threonine residues via oxygen linkages. Differences in monosaccharide composition, linkage between monosaccharides, branching structures, and others, give rise to a vast diversity of glycans that can act as a key regulatory mechanism, controlling both physiological and pathological processes [99]. *O*-GlcNAc is a unique post-translation modification, where GlcNAc is added to serines or threonines of intracellular proteins. Being reversible (OGT, *O*-linked *N*-acetylglucosamine transferase, adds the GlcNAc monosaccharide, and OGA, *O*-GlcNAcase, removes it) and shown to compete with phosphorylation modifications, it is a major regulator of cellular biology [100].

Alt-text: Box 1

Now is the time to crack the glycome code of T and B cells in immune development, which can give rise to a wealth of novel biological and mechanistic information. This can further lead to an understanding of when, why, and how glycosylation affects immune responses with putative consequences on the development of immune-associated diseases, including infection, autoimmunity, and cancer.

## The step-wise regulatory functions of glycans along T cell development

T cells are one of the key players of the adaptive immune response, simultaneously controlling multiple insults in the host and maintaining immune homeostasis. T cell development is one of the major physiological processes occurring in complex jawed vertebrates, which enables the formation of a proper repertoire of T cell receptors (TCRs), which are fundamental in adaptive immune reactions [11].

### Glycans in thymus seeding

**Figure 1:**
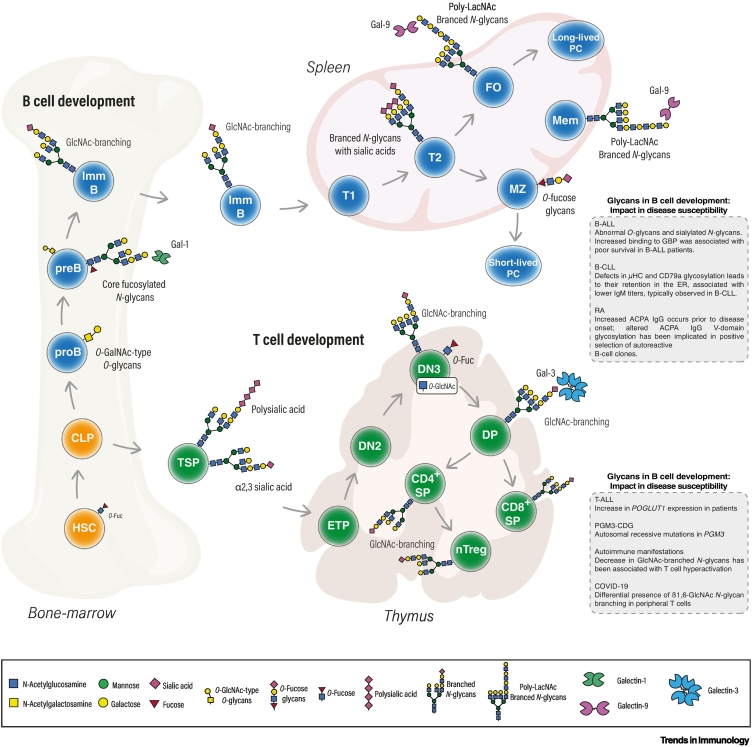
Key figure. Glycan dynamics and functions during mammalian T and B cell development. The development of the lymphoid lineage begins in the bone marrow (BM), where hematopoietic stem cells (HSCs) give rise to common lymphocyte progenitors (CLPs). CLPs either migrate into the thymus to generate T cells or begin the initial steps of B cell development in the BM, to be further differentiated in the spleen. Throughout, several post-translational modifications by glycosylation in key molecules and receptors of developing T and B cells occur that strictly regulate the survival, localization, inter-cellular interactions, and decision checkpoints that allow developmental progression. Defects in B and T cell development, in part due to changes in glycome composition, have been related to several human diseases (upper and bottom right boxes, respectively), including the development of cancers such as leukemias [B cell precursor acute lymphoblastic leukemia (B-ALL), B cell chronic lymphocytic leukemia (B-CLL), T cell acute lymphoblastic leukemia (T-ALL)], the onset of congenital disorders of glycosylation (CDGs), but also autoimmune diseases [such as rheumatoid arthritis (RA)] and infectious diseases such as coronavirus disease 2019 (COVID-19; see main text for references). Abbreviations: ACPA, anti-citrullinated protein antibody; DN, double negative; DP, double positive; FO, follicular; Gal, galectin; GBP, glycan-binding proteins; GlcNAc, *N*-acetylglucosamine; μHC, μ-heavy chain; MZ, marginal zone; PC, plasma cell; pre-B cell, precursor B cell; pro-B cell, progenitor B cell; SP, single positive; T1/T2, transitional stage 1 or 2; TSP, thymic seeding progenitor.

Most mammalian hematopoietic cell lineages develop in the bone marrow, derived from several differentiation events, and at the ‘top’ of the ‘differentiation tree’ the hematopoietic stem cell (HSC) progenitor pool is placed (Figure 1, Key figure) [11]. T cells, however, have an unique environment for development that is set in the thymus, which has to be reached by a hematopoietic progenitor, egressing from the bone marrow into the bloodstream. The cellular subset that represents the supply of progenitors to the thymus is composed of thymic seeding progenitor (TSP) cells [11]. At this stage, the interaction of P-selectin, a glycan-recognizing receptor expressed by the thymic endothelium, and its ligand, the P-selectin glycoprotein ligand-1 (PSGL-1), is required for thymic homing [12]. The glycosylation profile of PSGL-1 has been reported to be essential for its binding to P-selectin *in vitro* [13]. Mice deficient in core 2 *O*-glycan structures displayed impaired leukocyte recruitment to inflamed tissues, dependent on P-selectin binding [14]. In fact, mice lacking fucosyltransferase 4 (FUT4), fucosyltransferase 7 (FUT7) [15], or ST3 ß-galactoside α-2,3-sialyltransferase 4 (ST3GAL4), showed impaired TSP homing to the thymus, with no alteration in bone marrow engraftment relative to wild type mice [16]. Moreover, in the α2,8-sialyltransferase *St8Sia4*^–/–^ mouse model, reduced thymic cellularity and deficient thymic immigration by hematopoietic progenitors was reported relative to wild-type mice, suggesting that polysialic structures are involved in thymic colonization [17]. Together, this evidence supports a prominent role of glycans in thymus seeding, which is one of the earliest events in T cell development.

### Glycans in T cell lineage commitment

After TSP thymic entry, these cells develop into early thymocyte progenitors, a subset of the CD4^–^CD8^–^ double-negative (DN) population, which can give rise to multiple hematopoietic lineages [10], including myeloid ones [18] (Figure 1). The **Notch** (see Glossary) signaling pathway is essential for T cell lineage commitment of these immature thymocytes [19]. The genetic depletion of the *Notch1* receptor showed that it is required for murine T cell lineage specification [20]. Notch receptors are covered with a multitude of glycan structures, ranging from *O*-linked Fuc (*O*-fucose), *O*-Glc (*O*-glucose), extracellular *O*-GlcNac (*N*-acetylglucosamine), as well as *N*-glycans [21]. Within these several glycan structures, *O*-fucosylation in epidermal growth factor-like (EGF-like) repeats stand out because of their regulatory role in membrane trafficking of Notch receptors, observed in HEK293T cells [22], and in establishing differential affinities for Notch ligands [23]. Of note, mice that lack *Pofut1* (which encodes POFUT1, the fucosyltransferase responsible for the *O*-Fuc addition in the EGF-like repeats of Notch), in conditional knockout model induced by *Mx*^Cre^, show increased numbers of B cells in the thymus and impaired T cell development relative to controls [24]. These initial fucose monosaccharides are used by the lunatic, manic, and radical fringe glycosyltransferases, which transfer *N*-acetylglucosamine (GlcNAc) to *O*-fucose glycans, in a ß1,3 linkage [23]. The role of lunatic fringe, encoded by *Lfng* in T cell lineage commitment, has been the focus of several studies. An early RNA *in situ* hybridization study identified high *Lfng* expression in the murine thymic medulla and not in the cortex, where some DN subsets reside [25]. In fact, *Lfng*-overexpressing CD4^+^CD8^+^ double-positive (DP) murine thymocytes blocked T cell-oriented TSP development, through competition to functionally limiting delta-like ligands (DLLs), targets for Notch1 [26]. In addition, a mouse model of triple fringe deficiency showed reduced binding of Notch to DLL ligands, namely DLL-4, altering the frequencies of several T cell subsets in the thymus [27]. This body of work highlights the role of glycans, namely the Notch1 receptor, as regulators of T cell lineage commitment in the thymic microenvironment, at least in mice.

### Glycans in ß-selection

Once DN2 thymocytes commit to the T cell lineage, the progression to the DN3 stage occurs [10], where a decisive developmental checkpoint is placed. Through the action of the recombination-activation genes (RAG) complex, chromosomal rearrangements of the *Tcrb* gene are attempted and, upon a productive event, the expression of a functional pre-TCR (TCRß chain and pre-Tα) complex ensues [28]. Together with Notch and IL-7, pre-TCR signaling cascades initiate TCRß chain selection by inducing the transition to the DN4 stage, with multiple rounds of cell division, leading to the rise of the most abundant thymocyte population: the DP thymocytes (Figure 1). Our group reported that a deficiency in the expression of complex branched *N*-glycans in a mouse model of *Mgat1*-deficiency in DN2 thymocytes and onwards, using a *Rag1*^Cre^ line, resulted in abnormal expression of high-mannose *N-*glycans that was associated with defects in ß-selection [10]. In this model, the deletion of *Mgat1*, encoding the first GlcNAc transferase in *N*-glycan branches formation, blocked the glycosylation pathway, only allowing the formation of high-mannose structures in thymocytes. We observed that highly mannosylated DN3 cells had lower amounts of CD127 (IL-7 receptor α chain) and CD25 (IL-2 receptor), which contributed to defects in the generation of ß-selected DN3 thymocytes.

The glycome signature can be decoded and read by complementary glycan-binding proteins (GBPs) expressed or secreted by the immune system that translate glycan recognition into function. Galectins, C-type lectins, and Siglecs are the classical examples of GBPs through which glycans can contribute to the modulation of immune responses. Galectins play an important role in T cell development. Specifically, in galectin-3 (Gal-3)-deficient mice, DN3 cell numbers were found to be reduced, as were their proliferation rates, indicating a deficiency in ß-selection [29]. The ß-selection checkpoint has also been known to drive (and require) metabolic alterations in DN3 thymocytes [30]. In fact, the import of glucose and glutamine was increased in ß-selected thymocytes, with a parallel increase in protein *O*-GlcNAcylation (Box 1) [31]. In the same study, DN4-specific conditional knockout of *Ogt* (encoding the OGT enzyme, which is responsible for *O*-GlcNAcylation) led to a failure in the clonal expansion of those cells, resulting in decreased DP thymocyte numbers relative to wild-type mice [31]. The UDP-GlcNAc metabolite is a common substrate for *O*-GlcNAcylation and for *N*-glycan branching. Being a product of the hexosamine biosynthetic pathway, its *de novo* synthesis is regulated by a rate-limiting reaction catalyzed by the glutamine-fructose-6-phosphate transaminase 1 (GFAT1) enzyme. Deletion of the *Gfat1* gene at the DN2 stage recently showed increased presence of mannosylated *N*-glycans and decreased *O*-GlcNAcylation, which were associated with increased DN3 and decreased DP cell numbers, further indicating defects in ß-selected cell expansion. This suggested a requirement for both GlcNAc-branched *N*-glycans and *O*-GlcNAcylation for this checkpoint [32]. Collectively, the evidence highlights the regulatory power of glycans in ß-selection.

### Glycans in DP selection

ß-selected DN4 murine thymocytes undergo several rounds of cellular division, upregulating surface expression of CD8 [found in the transitional population of immature CD8^+^ single-positive (SP) cells] and finally CD4^+^, reaching the DP stage, where mature TCRs (composed by TCRß and TCRα chains) are generated (Figure 1) [33]. The newly formed TCRs are probed for their recognition potential in two selection steps by thymic epithelial cells (TECs). These cells, as a whole population, can present every peptide in the genome in MHC-I or MHC-II [34]. This feature enables the screening of TCRs by their specificity and binding strength [33]. One important aspect that remains unknown is the potential role of glycosylation of TECs in fine-tuning T cell selection, a relevant concept that is worth exploring in immunity.

The first selection step of DP thymocytes is termed ‘positive selection’, where cells that express competent TCRs (capable of recognizing peptide/MHC ligands) receive survival signals [33]. There, DPs also acquire MHC specificity, thus committing to the CD4^+^ or CD8^+^ SP lineage [35]. In the aforementioned model of *Gfat1* deficiency (with increased presence of mannosylated *N*-glycans), an overall decrease in TCRß surface expression in thymocytes was observed, which was accompanied by lower amounts of surface CD5 (proxy of TCR signaling) [32]. Moreover, CD4^+^ and CD8^+^ SP absolute numbers were decreased relative to littermate controls, showcasing the effect of UDP-GlcNAc deficiency in DP selection steps [32]; this aligns with previous observations by us and others on the role of *N*-glycan branching in DP selection, which is known to tune TCR signaling thresholds and thus DP selection [10,36]. In addition, the amounts of *O*-GlcNAcylation were shown to increase in positively selected cells, and the deletion of *Ogt* in DPs led to failure in positive selection, with a marked deficiency in mature SP populations, indicating a requirement for specific *O*-GlcNAc modifications in intracellular protein [31].

Positively selected cells then undergo ‘negative selection’, the second step, where thymocytes that display high levels of TCR signaling are eliminated, due to their autoreactive potential [33]. As the positive and negative selection processes require efficient TCR signaling, specific glycans are involved in this developmental checkpoint. For example, in a murine model of fucosyltransferase 8 (FUT8, responsible for core fucosylation of *N*-glycans) deficiency, positive selection was impaired and the frequency of mature SP thymocytes was decreased compared with wild-type mice. In fact, TCR signaling was attenuated in *Fut8*^–/–^ murine thymocytes, suggesting a role for core-fucosylated *N*-glycans in mediating positive selection [37]. Complex branched *N*-glycans can also regulate TCR clustering, mediating cellular thresholds for activation [38]. Branched *N*-glycans can also regulate the range of TCR signaling strengths in positive selection, through the promotion of CD4^+^ or CD8^+^ surface expression. Moreover, the role of *N*-glycans in the tuning of TCR signaling and clustering was also described in the context of negative selection, using murine models of conditional deletion of *Mgat1* and *Mgat2* [36]. In a recent study, we also observed higher rates of negative selection in *Mgat1*-deficent thymocytes (expressing mannosylated *N*-glycans), as well as defects in TCR repertoire variability both in CD4^+^ and CD8^+^ SP cells; this suggested that the tuning of TCR signaling strengths by *N*-glycans in thymocytes could affect peripheral tolerance [10]. Of note, in *Mgat2*-deficent thymocytes, the developmental defects were not remarkable when compared with littermate controls and *Mgat1* deficiency models [10,36]. In fact, the existence of a compensatory mechanism for the glycosylation pathway has been reported: in the absence of glycosyltransferases responsible for *N*-glycan branching, there is a remodeling of Golgi compartments, leading to hyperelongation of existing GlcNAc-branches and, thus, to a functional compensatory effect [39]. Furthermore, the role of branched *N*-glycans in the regulation of TCR clustering is also highlighted by the binding of TCR glycans to Gal-3, resulting in the formation of a molecular lattice that controls the threshold for T cell activation [40]. Indeed, *Gal3*^–/–^ mice showed increased amounts of apoptosis in DP thymocytes compared with wild-type mice, suggesting that Gal-3 is important in controlling cell death during selection and supporting a general role for galectins in T cell development [38].

From another angle, **naturally occurring Tregs (nTregs)** are generated in the thymus from a pool of CD4^+^ SPs with high amounts of TCR signaling, close to the threshold of negative selection [41]. In agreement with the observation that negative selection is promoted in the absence of branching *N*-glycans [10,36], we recently observed that in the absence of branched *N*-glycans (*Mgat1* deficiency) the generation of nTregs was severely impaired [10].

In addition, we also reported that murine γδT cell development and activity were influenced by the glyco-reprogramming of thymocytes, namely in the conditional *Mgat1* knockout. We observed that in the absence of complex *N*-glycans, increased frequencies and levels of activation of thymic γδT cells were maintained in the periphery, leading to autoimmune characteristics of these mice when compared with littermate controls [10]. For *Gfat1* deficiency in a thymocyte model (with increased presence of mannosylated *N*-glycans), increased frequencies of γδT cells and decreased repertoire variability were noted relative to wild-type controls [32]. Both studies highlight a major role of glycans in γδT cell development and function, certainly deserving further attention.

Altogether, there is a solid body of evidence on the role of glycans in regulating T cell development, which points towards the need to apply novel technologies, such as glyco-proteomics, to the study of thymocyte development. Furthermore, with the use of lectins (proteins that recognize specific glycan structures), we have also reported that human thymocytes display glyco-profile alterations during development; thus, this highlights the need to focus on human samples to envision clinical translation.

## The regulatory functions of glycans during B cell development

B lymphocytes are fundamental players in the adaptive immune system, responsible for producing the entire repertoire of antibodies with antigen specificity in a host. B cell development is a highly regulated physiological process that takes place in the bone marrow and the spleen [42,43]. As in thymocytes, developing B cells undergo **somatic gene rearrangement** in immunoglobulin (Ig) loci; these cells are selected to ensure a self-tolerant antibody repertoire. B cell development is crucial to ensure proper immune functions throughout the life of the organism.

### Glycans in the progenitor B (pro-B) to precursor B (pre-B) cell transition

The transition from pro-B cell to pre-B cell requires rearrangement of the Ig μ-heavy chain (μHC) locus, resulting in the formation of the pre-B cell receptor (BCR), still devoid of antigen specificity, but triggering a signaling cascade that drives the rapid clonal expansion of pre-B cells (Figure 1) [8,44]. *N-*linked glycosylation on μHC was shown to be essential for pre-BCR assembly [8]. Specifically, core fucosylation, catalyzed by FUT8 activity, plays a fundament role in pre-B cell generation [9,45,46]. Indeed, *Fut8*^–/–^ mice show abnormalities in pre-B cell generation. In addition, *in vivo* (*Fut8*^–/–^ mice) and *in vitro* studies using 70Z/3 cells transfected with a siRNA targeting *Fut8*, revealed that core fucosylation of μHC was crucial for its interaction with the λ5 light chain, mediating the assembly of the pre-BCR; this ultimately affected pre-BCR intracellular signaling and proliferation [9]. Additionally, loss of core fucosylation, by using *Fut8*^–/–^ mice, impaired the interaction between the very late antigen 4 (VLA-4 integrin) on pre-B cells and the vascular cell adhesion molecule 1 (VCAM-1) on stromal cells, leading to a defect on the colony expansion of pre-B cells [45]. Such evidence suggests that core fucose is essential for pre-B cell development and function, through different mechanisms. Moreover, galectin-1 (Gal-1), produced by bone marrow stromal cells, was found to interact with glycosylated forms of pre-B cell integrins, promoting pre-BCR clustering and, thus, enabled efficient B cell development of normal pre-B cells [47., 48., 49.]. Moreover, Gal-3 deletion resulted in abnormal numbers of several B cell subsets [50]; also, Gal-3 acted as an inhibitor of stromal-derived IL-7 and Notch/Jagged-1 signaling, suggesting that its expression might act on bone marrow stromal cells modulating B cell development, although this remains to be further investigated [50]. Together, these studies highlight the importance of glycans as modulators during this highly controlled phase of B cell development.

### Glycans in immature B cell generation and selection

After clonal expansion of pre-B cells, signaling activation of the pre-BCR leads to DNA rearrangement of the Ig μ-light chain (μLC) locus to form a mature BCR that progresses into immature B cells [51]. Immature B cells enter a selection process in the bone marrow to assess their autoreactive potential. If BCR binds a self-antigen, immature B cells die by negative selection, whereas cells that display a tolerant BCR are positively selected, leading to central B cell tolerance (Figure 1) [52]. *N*-glycan branching seems to be required for a proper B cell selection. Specifically, using a mouse model of B cell-specific deletion of the *Mgat1* gene (*Mgat1*^f/f^*Cd19*^Cre^) rendering branched *N*-glycan deficiency in B cells, decreased surface expression of the BCR co-receptor CD19 was observed, which inhibited positive selection. Moreover, the nerve growth factor IB (Nur77, related to B cell activation) was upregulated in immature B cells in *Mgat1*^f/f^*Cd19*^Cre^ mice, indicating the prominent role of complex *N*-glycans in defining B cell thresholds, similar to T cells [53]. In a B cell-specific *Cosmc* deficiency model, (*Cosmc*^f/f^*Mb1*^Cre^ mice) in which *O*-glycans were truncated, pro- and pre-B cell populations were decreased relative to wild-type controls. By contrast, immature B cell numbers were increased in the bone marrow, possibly related to the defects in B cell peripheral homing observed in these mice [54]. This suggested that the progression to an immature B cell stage is regulated by both *N*- and *O*-glycans.

### The role of glycans in bone marrow egress and B cell peripheral maturation

After egressing from the bone marrow, immature B cells home to the spleen, where they mature in **transitional stages (T1/2)** until reaching the mature B cell stage (Figure 1) [55]. Again, by using *Mgat1*^f/f^*Cd19*^Cre^ mice, branched *N*-glycan structures were reported to be essential for the survival of T2 transitional peripheral B cells, but not mature B cells. Indeed, branched *N*-glycans were important in the maintenance of homeostatic pre-BCR/BCR signaling threshold boundaries, allowing positive selection by inhibiting both death by neglect and negative B cell selection [53]. This suggested that branched *N-*glycans might act as fundamental players in this regulatory step, which can further affect peripheral tolerance and the development of immunopathological diseases.

Sialic acids also have a major impact on B lymphocyte function. In fact, blocking the synthesis of these terminal carbohydrate structures, specifically on B cells by using *Cmas*^f/f^*Mb1*^Cre^ mice, resulted in a remarkable reduction in immature B cells and an almost complete absence of mature B cells, which were linked to the triggering of an apoptosis-mediated mechanism [56]. Additionally, extrinsic sialylation by extracellular sialyltransferase ST6GAL1, which generates α2,6-linked sialic acids in glycoconjugates, was reported as an important pro-survival factor guiding B cell development [57]. Indeed, mice harboring *St6gal1*-deficient B cells revealed that IgG sialylation occurred independently of the B cell secretory pathway, supporting the idea that sialylation might be regulated in the extracellular environment [58]. Later, using B cell hybridomas and *ex vivo* murine B cells, the same group described that IgG in B cells localizes to regions that are rich in core fucosylation, thus having limited exposure to and modification with ST6Gal1; this suggested that B cell-expressed ST6Gal1 might be dispensable for IgG sialylation *in vivo* [59]. However, further studies are needed to understand the underlying mechanism behind IgG trafficking and glycosylation.

In the spleen, mature or naive B cells commit to the **marginal zone (MZ)** or **follicular (FO)** zone before differentiating into memory B cells or plasma cells upon antigen activation [60]. B cell-intrinsic ST6Gal1 was shown *in vivo* to be required for MZ B cell development [57]. Furthermore, sialic-binding Ig-type lectin (Siglec-2), CD22, a B cell inhibitory co-receptor that binds to sialic acids either in *cis* or *trans* linkage, has influenced the number of MZ B cells in the spleen through the regulation of B cell Ca^2+^ signaling [61]. *O*-Fucose glycans also appear to be pivotal for the efficient binding of Notch receptors with their canonical Notch ligands. For instance, inactivation of *Pofut1* using *Mx*^*Cre*^*Pofut1*^f/f^ mice, gave rise to aberrant MZ B cell formation, myeloid hyperplasia, and thymic hypoplasia. *Pofut1* deficiency abolished the binding of Notch receptors and ligands, suppressing downstream activation. These data support the crucial role of *O-*fucose glycans in Notch regulation of hematopoietic homeostasis [24].

Taken together, the body of evidence described highlights a major role of glycans in B cell bone marrow egress and peripheral maturation.

### Glycans in mature B cell activation and GC formation

Once in lymphoid organs, mature naive B cells will recognize antigen and become activated. Antigen-induced B cell activation and differentiation then gives rise to the GC reaction (Figure 1). The GC is characterized by clonal expansion, **class switch recombination (CSR)**, **somatic hypermutation (SHM)**, and selection for increased affinity of a BCR with a unique antigenic epitope (**affinity maturation**). In the GC, B cells display changes in glycan profiles for cell surface glycoproteins. These distinct changes are widely used to identify GC B cells. For instance, the glycan epitope α2,6-linked *N*-acetylneuraminic acid (Neu5Ac) is recognized by the GL-7 antibody, which is used to identify GC B cells [62]. The increase in Neu5Ac results from the downregulation of the CMAH enzyme (CMP sialic acid hydroxylase), which is responsible for the hydroxylation of sialic acid, preferentially expressed on murine naive B cells [63]. This switch has a profound effect on CD22 function, which plays a role in the homeostasis of GC B cells. In fact, this glycosylation change is crucial for the maintenance of antigen-specific GC B cells, the generation of plasma cells and memory B cells, as well as affinity maturation of antibodies [64]. Humans have an inactive *CMAH* and there is similar downregulation of CD22 ligands in human B cells, but the ligand structure and enzymes regulating the change differ, showing that this downregulation is an evolutionarily conserved mechanism in both murine and human species, yet involving different enzymes [63,65]. Furthermore, naive and memory B cells from human tonsils were reported to express branched *N*-glycans at the cell surface, elongated with poly-*N*-acetyllactosamine (poly-LacNAc) structures that served as high affinity binding sites to galectins, such as Gal-9 [66]. Moreover, Gal-9 binding was documented to regulate BCR signaling, inhibiting naive B cell activation [66]. During homeostasis, B cell differentiation into memory B cells or plasma cells was accompanied by changes in *O*-glycosylation, such as loss of *O-*glycan structures, including loss of extended core 1 and core 2 *O-*glycans, regulated by ST3GAL1 and GCNT1 enzymes [67].

Taken together, the role of glycans during B cell development is far from being fully elucidated; however, it has the potential to unlock novel mechanisms underlying humoral immune responses. These might be leveraged to further understand the functional outcomes of disease development, as discussed later.

## Alterations in glycosylation pathways during T and B cell development: impact on disease susceptibility

Defects in the normal development of T and B cells have been related to several human diseases. As both processes entail chromosomal rearrangements and/or the induction of genomic mutations, in combination with failed developmental checkpoints, the risk of malignancy-driving events is quite high. Changes in the glycosylation profile of lymphocytic pre-malignant cells may promote cellular transformation, as it occurs in other cancer types [68]. In T cell acute lymphoblastic leukemia (T-ALL) patients, increased expression of *POGLUT1* (*O*-glycosyltransferase 1; responsible for *O*-glucose additions in NOTCH1) was noted when compared with healthy controls [69]. In a murine model of T-ALL, with a *Pten* deletion (gene encoding an important negative regulator of cell proliferation [70]) in an *Ogt*-deficient background in DN2 cells, an essential role for *O*-GlcNAcylation was observed; indeed, in *Ogt*-deficient thymocytes, malignant transformation was decreased [31]. In B cell precursor ALL (B-ALL) patients, increased *O-*glycome complexity as well as overall increased sialylated *N-*glycans were observed, using glyco-proteomics, when compared with normal pre-B cells [71]. In addition, B-ALL patients exhibited increased RNA and protein expression of GCNT1 and GALNT7 enzymes [71]. Moreover, ALL cells were reported to be more targeted by GBPs expressed in innate immune cells, such as C-type lectins DC-SIGN and L-SIGN (as evidenced from increased binding of these cells using recombinant GBPs); this is relevant as it was associated with poor B-ALL patient survival [72]. Furthermore, in B cell chronic lymphocytic leukemia (B-CLL), lower IgM expression, when compared with healthy controls, resulted from defects in glycosylation of μHC and CD79a, leading to their retention in the endoplasmic reticulum (ER), possibly leading to the lower IgM expression [73]. Together, this evidence highlights the possibility that certain perturbations in glycosylation pathways may be connected with risk of malignancy for lymphoid tumors, supporting the relevance of glycans in immune homeostasis. However, the precise roles of glycans and the mechanisms underlying leukemic transformation remain far from being understood. As the knowledge regarding the regulatory role of these glycan structures in developmental processes is increased, the opportunities for the discovery of novel malignant activators and/or inhibitors might present themselves.

From another angle, congenital disorders of glycosylation (CDGs) is a group of genetic diseases with mutation in glycogenes that often present hypoglycosylation states (or defects in one or more glycosylation pathways) and can be associated with the development of some immune phenotypes, such as primary immunodeficiencies or autoimmunity [74]. For instance, PGM3-CDG is a disorder characterized by autosomal recessive mutations of the *PGM3* gene encoding phosphoglycomutase 3, an enzyme that catalyzes the conversion of GlycNAc-6-phosphate into the UDP-GlcNAc precursor molecule GlcNAc-1-phosphate. PGM3-CDG patients show CD4^+^ and CD8^+^ T cell lymphopenia [75], which is, thus far, only treatable with HSC transplantation. Indeed, HSC transplantation has been reported to correct disease symptoms, such as recurrent infections [76,77].

Defects in glycosylation have also been associated with autoimmunity. In a recent study, we observed increased amounts of spontaneous inflammation in the colon and kidney tissues of *Rag1*^Cre^*Mgat1*^fl/fl^ mice (where thymocytes and T cells only expressed abnormal high-mannose *N*-glycans), relative to littermate controls [10]. Accordingly, *Mgat5*^–/–^ mice, which lack β1,6 GlcNAc-branched *N*-glycans, displayed the spontaneous development of glomerulonephritis phenotypes [38] and a Lupus-like syndrome with proteinuria and body weight changes [78], as also observed in *Man2a1*^–/–^ mice (with a deficiency in alpha mannosidase II enzyme, responsible for the removal of mannose monosaccharides) [79]. In *Mgat5*^–/–^ mice, the hyperactivation of T cells, mediated by decreased thresholds for TCR activation imposed by the absence of complex *N*-glycans, was shown to be the major driver of immunopathogenesis of chronic inflammation and autoimmunity [80]. In fact, inflammatory bowel disease (IBD) patients showed decreased amounts of ß1,6-GlcNAc *N*-glycan branching in T cells, which was associated with mucosal T cell hyperactivation and disease severity [39,80,81]. Moreover, genetic variants of the *MGAT5* glycogene were described in ulcerative colitis patients and found to be associated with low transcription amounts of *MGAT5*, and agalactosylation of circulating IgGs, often associated with proinflammatory phenotypes [82]. When comparing nonvaccinated, infected individuals with coronavirus disease 2019 (COVID-19; as well as asymptomatic ones) versus non-infected, different branched *N*-glycan expression amounts in peripheral CD8^+^ T cells were reported [83]; this suggested that initial stages of infection response might involve the remodeling of the glycoprofiles of circulating T cells, although this remains to be investigated. In rheumatoid arthritis (RA), the presence of variable domain glycans (VDGs) has been described in B cell-producing anti-citrullinated protein antibodies (ACPAs) due to SHM events, which results in the selective introduction of *N*-linked glycosylation sites in the BCR; this is presumed to act as an important driver of RA development [84,85]. Of note, B cell-producing ACPA contains biantennary *N*-glycans in the V-domain with one or two terminal sialic acid residues and one core fucose [86]; this is relevant as it has been proposed to be involved in the positive selection of autoreactive B cell clones, influencing antigen binding [87]. ACPA B cell responses mature prior to the onset of disease [88]; however, the precise contribution of glycans as ‘a key switch’ of B cell autoreactivity in RA deserves rigorous investigation.

The growing body of evidence on the role of glycans in modulating the effects of circulating antibodies, such as IgG, in disease has gained momentum in previous years [89]. In fact, The Fc domain of IgG contains a single, highly conserved, *N*-glycosylation site that carries complex *N*-glycans; these regulate its binding to Fc receptors and, therefore, seems to contribute to its pro- or anti-inflammatory function. For instance, the IgG Fc *N*-glycome has been associated with several diseases, such as RA [90], IBD [82], and type 2 diabetes [91]. In COVID-19 patients, we and others reported that IgG glycans, namely their levels of fucosylation, galactosylation, and sialylation, were differentially present in patients with severe disease when compared with those with mild disease; this also highlighted a glycosylation-based feature of immediate infection responses, at least for severe acute respiratory syndrome coronavirus 2 (SARS-CoV2) [92., 93., 94.].

Taken together, the precise relationship between glycan influence on T and B cell differentiation events and disease susceptibility is an exciting research area that should be further explored because some of the features of adaptive immune cells are imprinted during development.

## Concluding remarks

Although usually underappreciated, exciting findings in the last couple of years have underscored the essential contributions of protein glycosylation in fundamental cellular and molecular mechanisms underlying immune tolerance and homeostasis. These have been changing the conceptual landscape of the pathogenesis of immune-related diseases. Technological advances in the past decade, such as glyco-proteomics, have brought us to the level where reliable and extensive glycan analysis can be routinely performed on a large number of samples and even single-cell glycomics is within reach [95]. **Biorthogonal chemistry (click-chemistry)**, recently distinguished by the Nobel Prize in Chemistry 2022, has proven to be an essential tool for understanding the structures, localization, and biological functions of glycans at cellular surfaces, allowing non-invasive imaging of glycans in a living system [96]. Statistical and bioinformatics tools for the integration of glycomics with other omics data, such as transcriptomics, metabolomics, and immune profile, can allow a deeper glyco-immune-proteome mapping and may reveal a more complete picture of glycans, ideally unlocking the sugar code of immunity in a structure–function relationship. In this opinion article, we raised the hypothesis that the developmental regulation of the glycome occurs alongside and may impact T and B cell development. We posit that this information can be leveraged to further understand basic principles of immunity and assess clinical implications (see Outstanding questions). Future research should focus on applying novel technologies to decipher the glycome for developing T and B cells, gain further insights into glycan–immune receptor pairs (glycans-GBP), and explore their mechanistic impact during homeostasis and disease.

Clinician’s corner Certain leukemic patients display differential glycogene expression, often related to sustained malignancy-driving signaling cascades [69,71,73].

Congenital disorders of glycosylation often display immunological phenotypes, including immunodeficiency and autoimmunity [74., 75., 76., 77.].

The glycoprofile of peripheral T and B cells can be associated with certain inflammatory conditions or disease severity [39,80., 81., 82., 83.].

The presence of variable domain glycans on B cell-producing antibodies has been reported to be predictive of autoimmune development for certain diseases [84., 85., 86., 87., 88.].

Circulating IgG glycans have been associated with onset and severity for certain diseases, such as type 2 diabetes and COVID-19 [91., 92., 93., 94.], although further investigations are necessary.

Alt-text: Clinician’s corner

Outstanding questions What is the precise glycome composition of developing T and B cells? Novel comprehensive technologies, combining glycomics, glycotranscriptomics, and glycoproteomics, should be employed to address this key question, achieving glycome mapping across T and B cell development.

How do glycans precisely contribute to the regulation of key immune receptors during T and B cell development? The use of conditional mouse models to perturb specific glycan structures in each developmental stage and for specific receptors might reveal mechanisms by which glycans can modulate T and B cell development and the functional immune consequences thereof.

Are glycans involved in immune disease susceptibility during lymphocyte development? Glycoengineered murine models combined with glycome determination in specific patient samples might provide further information on how glycosylation deficiencies in developing T and B cells might associate with disease risks. One might envision the identification of novel putative disease biomarkers for prediction and candidate therapeutic targets.

How do changes in glycosylation profiles arise and which biological events/mechanisms are regulating such changes? Genetic depletion and mutant models impose forced glycan changes that result in limited/controlled effects on specific cells or tissues under certain normal or pathological conditions. Multi-disciplinary approaches combining *in vitro*, *ex vivo*, and *in vivo* animal models, as well as human samples, should be employed to address when, why, and how these glycosylation changes occur.

Alt-text: Outstanding questions

## Glossary

**Affinity maturation**: selection for increased BCR affinity for a unique antigen epitope. SHM does not distinguish between favorable and unfavorable mutations; thus, a selection process for antigen binding occurs in the light zone of the germinal center to select B cells that produce the highest affinity antibodies.
**Biorthogonal chemistry (click-chemistry)**: chemical reactions not found in, but which can be set within living systems.
**Class switch recombination (CSR)**: DNA recombination process of the immunoglobulin (Ig) constant region after T cell priming.
**Follicular (FO)**: most mature B cells are recirculating cells that home to B cell follicles in secondary lymphoid organs. Follicles are adjacent structures adjacent to T cell zones, which allows the activation of FO B cells in a T cell-dependent immune response.
**Marginal zone (MZ)**: well-defined anatomical compartment of the spleen, located at the outer limit of the white pulp. The main resident cells are naive MZ-B cells.
**Naturally occurring Tregs (nTregs)**: naturally occurring regulatory T cells, generated in the thymus, derived from a pool of CD4^+^ SP cells with high amounts of activation, close to the threshold of negative selection.
**Notch**: (NOTCH1-4 in mammals) evolutionary conserved heterodimeric glycoprotein receptors, which can bind to two families of Notch ligands.
**Somatic gene rearrangement**: also known as V(D)J DNA recombination, involves the rearrangement of the variable (V), diversity (D), and joining (J) gene segments in the heavy (H) chain locus and V and J gene segments in the light (L) chain locus of immunoglobulins.
**Somatic hypermutation (SHM)**: in germinal centers, point mutations in the antibody variable regions of both heavy and light chains.
**Transitional stages (T1/2)**: occur after initial V(D)J recombination and before the maturation of mature B cells. During the T1 stage, B cells migrate from the bone marrow to the spleen and undergo positive and BCR-mediated negative selection to eliminate self-reactive B cells. In the spleen, T2 B cells undergo additional selection processes and further differentiation until they reach the mature stage.
